# Optimized Method for Robust Transcriptome Profiling of Minute Tissues Using Laser Capture Microdissection and Low-Input RNA-Seq

**DOI:** 10.3389/fnmol.2017.00185

**Published:** 2017-06-13

**Authors:** Shannon Farris, Yu Wang, James M. Ward, Serena M. Dudek

**Affiliations:** ^1^Neurobiology Laboratory, National Institute of Environmental Health Sciences, National Institutes of HealthResearch Triangle Park, NC, United States; ^2^Cellular and Molecular Pathology, National Toxicology Program, National Institute of Environmental Health Sciences, National Institutes of HealthResearch Triangle Park, NC, United States; ^3^Integrative Bioinformatics, National Institute of Environmental Health Sciences, National Institutes of HealthResearch Triangle Park, NC, United States

**Keywords:** RNA-Seq, laser-capture microdissection, RNA quality, low-input, hippocampus, transcriptome

## Abstract

Obtaining high quality RNA from complex biological tissues, such as the brain, is needed for establishing high-fidelity cell-type specific transcriptomes. Although combining genetic labeling techniques with laser capture microdissection (LCM) is generally sufficient, concerns over RNA degradation and limited yields call into question results of many sequencing studies. Here we set out to address both of these issues by: (1) developing a fluorescence-assisted LCM protocol that yields high quality RNA from fresh-frozen tissues; and (2) determining a suitable RNA-Seq library generation method for limited amounts of RNA (1–5 ng total RNA). The latter focused on comparing commercially available kits able to produce libraries of sufficient concentration and complexity while limiting PCR amplification biases. We find that high quality RNA (RNA integrity number, RIN, >9) of sufficient concentration can be isolated from laser-captured material from thinly-sectioned tissues when digestion time and temperature are minimized. Furthermore, we found that library generation approaches that retain ribosomal RNA (rRNA) through cDNA library generation required fewer cycles of PCR, minimizing bias in the resulting libraries. Lastly, end stage depletion of rRNA prior to sequencing enriches for target RNAs, thereby increasing read depth and level of gene detection while decreasing sequencing costs. Here we describe our protocol for generating robust RNA-Seq libraries from laser-captured tissue and demonstrate that with this method, we obtain samples with RNA quality superior to the current standard in the LCM field, and show that low-input RNA-Seq kits that minimize PCR bias produce high fidelity sequencing metrics with less variability compared to current practices.

## Significance Statement

Transcriptome profiling of minute samples is critical for understanding complex tissues. Given the unstable nature of RNA, current methods have been unable to ensure that high quality RNA is obtained for subsequent sequencing applications. Here we compared commercially available low-input RNA isolation kits to evaluate RNA quality and low-input RNA-Seq library kits to evaluate the robustness of low-input library generation approaches. We find that high quality RNA can be extracted in sufficient quantity from thinly-sectioned laser-captured tissues when minimal digestion time and temperature are used. Furthermore, we found that library generation approaches that retain rRNA through cDNA library generation required fewer cycles of PCR, which minimized bias in the libraries and produced more uniform gene coverage with less variability than current practices. Combined, our methods enable high quality and robust transcriptome profiling from minute tissue samples.

## Introduction

Laser capture microdissection (LCM) is a microscopy-based technique developed to isolate select cell populations from complex, heterogeneous tissue. Using a thermoplastic polymer film that can be focally activated by infrared (IR) or ultraviolet (UV) lasers, discrete regions of tissue can be rapidly procured while retaining cellular morphology and location useful for identifying certain cell populations (Emmert-Buck et al., 1996; Bonner et al., 1997). The thermoplastic polymer can be heat-activated using either: (I) an IR laser onto a polymer coated cap to then lift a polymer-cell composite from the tissue; or (II) a UV laser to carve a border around the tissue of interest mounted onto a polymer slide that can be subsequently collected onto an adhesive cap (Emmert-Buck et al., 1996; Bonner et al., 1997; Espina et al., 2006). The UV laser LCM systems are well-suited for thick (up to 200 μm) tissues. However, the cells within the UV laser path are inevitably damaged, which adversely affects the quality of isolated DNA, RNA or proteins from small areas of interest (Espina et al., 2006). In contrast, the IR laser LCM systems use mild, transient focal heating of the thermoplastic polymer cap that produces minimal damage to DNA, mRNA or protein when brought into contact with the tissue (Bonner et al., 1997). Thus, understanding which LCM method is most appropriate for the experimental goals is essential. Here we report on our efforts to determine which LCM method was optimal for isolating RNA from discrete cell types in thinly sectioned (8 μm) fresh-frozen mouse tissues (brain and liver) for subsequent RNA-Seq.

RNA quality and quantity are two measures that can greatly affect the outcome of RNA-Seq studies (Adiconis et al., 2013; Gallego Romero et al., 2014). Existing cDNA library generation techniques deal with low quality or degraded RNA samples, such as random priming combined with various ribosomal RNA (rRNA) depletion methods (Tariq et al., 2011; Yi et al., 2011; Morlan et al., 2012; Ramsköld et al., 2012). However, usually these technologies require greater input amounts to achieve similar sequencing metrics obtained with high quality RNA. Because isolation of small cell populations using LCM typically results in minimal RNA yields, we set out to determine which LCM instrument and RNA extraction methods would yield the highest quality RNA. Previous studies have attempted to optimize obtaining high quality RNA from LCM (Wang et al., 2009, 2010; Cummings et al., 2011; Butler et al., 2016), however these studies report varying degrees of RNA quality for different types of tissues. We tested two commonly used LCM systems, one IR- and one UV-laser system, and determined that for small, thinly-sectioned fresh-frozen mouse sections the IR system resulted in higher quality RNA. In addition, we also tested whether different methods of RNA isolation had an impact on RNA quality and found that kits with minimal digestion time and temperature, such as 5 min at room temperature in the QIAGEN Micro RNeasy kit, resulted in superior quality RNA (RNA Integrity Number, RIN, 9–10, 10 being intact RNA) compared to longer digestion times at higher temperature, such as 30 min at 42°C in the Arcturus PicoPure RNA Isolation Kit (RIN 6–7), which is the current standard kit used in studies using LCM.

We next tested two commercially available low-input RNA-Seq library generation kits to determine which approach generated libraries of sufficient concentration while minimizing PCR amplification biases. Currently, the most frequently observed practice in the field is to amplify by PCR low-input RNA samples in order to generate sufficient input for cDNA library generation (Nichterwitz et al., 2016). This approach is expected to introduce PCR bias from non-uniform amplification driven by factors such as copy number (Coenen et al., 2015), transcript length (Oshlack and Wakefield, 2009) and GC nucleotide content (Lahens et al., 2014; van Dijk et al., 2014). As a result, the level of amplification products may poorly reflect the starting material. Two kits that have been previously identified as suitable for low-input RNA samples are the NuGEN Ovation RNA-Seq kit and the Clontech SMARTer kit (Tariq et al., 2011; Adiconis et al., 2013). Upon comparing these kits, we determined that library generation approaches that retain rRNA throughout cDNA generation, such as the NuGEN Ovation RNA-Seq system, require fewer cycles of PCR, likely due to preservation of yield prior to cDNA synthesis. The resulting libraries minimized PCR bias for low-input samples while maintaining high percent mapping, even gene coverage profiles, and low variability across replicates compared to current field standards.

## Materials and Methods

### Animals

Experiments were carried out in adult mice (see strains below) housed under a 12:12 light/dark cycle with access to food and water *ad libitum*. All procedures were approved by the Animal Care and Use Committee of NIEHS and were in accordance with the National Institutes of Health guidelines for care and use of animals.

### Tissue Preparation

For hippocampal sections: brains were harvested from adult male Amigo2-EGFP transgenic mice (25–35 g, 6–8 weeks) born from separate litters. The Amigo2-EGFP line was acquired from GENSAT (founder line LW244, RRID:MMRRC_033018-UCD) and bred for at least 10 generations onto C57bl6/n background. Mice were deeply anesthetized with Fatal Plus (50 mg/kg) before decapitation and swift removal of the brain (<2 min). Brains were bisected in the sagittal plane and individually flash frozen in 22 × 22 mm disposable cryomold (Polysciences, Inc., Warminster, PA, USA) filled with Tissue-Tek Optimal Cutting Temperature (OCT, Sakura Finetek) compound and then submerged in isopentane cooled to −20°C in a dry ice and ethanol slurry. For midbrain sections: adult C57bI6 mice were treated with a single systemic injection of LPS (5 mg/kg) or saline at 2 months old and a body weight of roughly 22–24 g for an unrelated study. After 10 months, brains were removed and immediately embedded in 4557 standard cryomold (Tissue-Tek^®^, Sakura) with OCT on dry ice. For liver sections: liver tissues were harvested from male C57bI6 mice (15–20 g, 6 weeks) and embedded as above for midbrain sections. Frozen tissue blocks were stored at −80°C until processing.

### Processing Brain Sections for LCM

**Figure 1:**
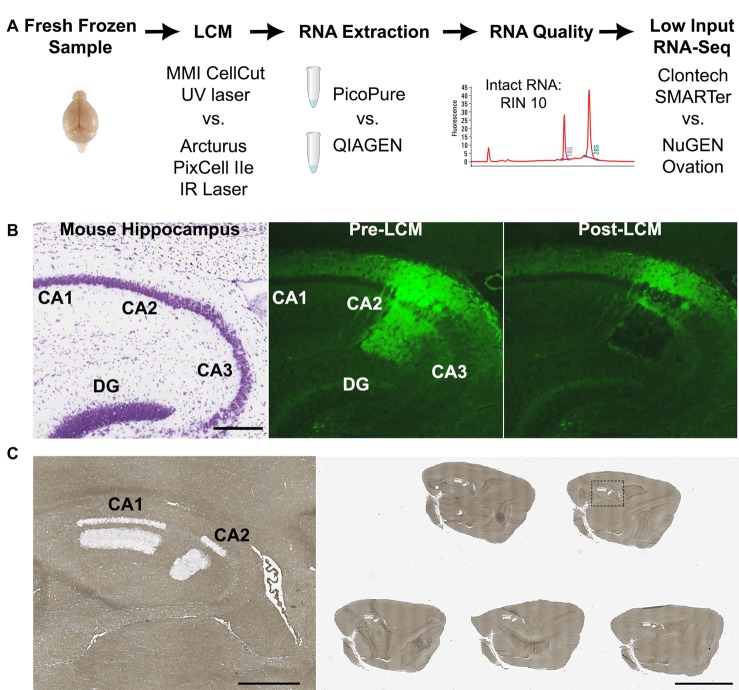
Laser capture-RNA-Seq workflow. **(A)** Schematic of steps that were optimized in the current study (LCM instrument, RNA extraction and RNA-Seq library kit). **(B)** Representative images of (left) nissl stained mouse hippocampus from Allen Brain Atlas, (middle) Amigo2-EGFP mouse hippocampus showing the fluorescently labeled CA2 cells and projections prior to LCM and (right) post LCM. **(C)** Representative image of a glass slide with five dehydrated mouse brain sections post-LCM of hippocampal subregions CA1 and CA2. On the left is a magnified image of the dashed box. Scale bars: 500 μm (**B,C** left) and 4 mm (**C** right). CA, cornus ammonis; DG, dentate gyrus; LCM, laser capture microdissection; RIN, RNA Integrity Number.

Frozen tissue blocks were transferred on dry ice to the cryostat (CM1850, Leica Microsystems) that was pre-set to –18°C and allowed to equilibrate for at least 10 min prior to cutting. To avoid contamination across samples, a clean paint brush was used instead of the roll plate. In order to accommodate five sections per slide, the frozen OCT blocks were trimmed on all sides using a razor blade, leaving 3–4 mm of OCT surrounding the tissue for handling with the paint brush. Eight micron thick sections were cut and mounted on either RNase-free Superfrost Plus glass slides (FISHER) for use with the PixCell instrument, or MicroDissect polyethylene terephthalate (PET) membrane single frame slides (ASEE, part # FS-LMD-M-50r) for use with the CellCut instrument. Prior to LCM, a few sections were stained with cresyl violet to verify coordinates (Dorsal Hippocampus: 1.00–2.00 mm lateral from midline in the sagittal plane) using the 1st edition Watson and Paxinos (2010). EGFP fluorescence was used to visualize the target cells (in this case hippocampal cornus ammonis 2 (CA2), see Figure 1B) with an inverted fluorescence microscope.

Five serial 8-micron thick sections from Amigo2-EGFP mouse brains were lined up in the cryostat and simultaneously mounted onto the slide (example slide shown in Figure 1C). An essential step was to mount all the sections on to the slide at once because picking up serial sections one by one or leaving sections at room temperature caused a reduction in fluorescence signal. After sections were mounted, the slide was immediately immersed in ice cold, RNase-free 95% ethanol for 30 s. Delaying fixation also reduced the fluorescence signal. Next, the slide was transferred to 75% ethanol for 30 s to remove the OCT. The slide was then gently swirled in the following solutions for 30 s each for sufficient dehydration: 95% ethanol, 2 × 100% ethanol. Swirling was particularly important in humid climates as insufficient dehydration affected both the fluorescence signal and ability to efficiently microdissect the tissue. For non-fluorescent midbrain and liver samples, 8-micron thick sections were fixed in ice cold 95% ethanol for 30 s, then transferred to 75% ethanol for 30 s to remove the OCT, as above. Slides were then stained with 2% cresyl violet in 75% ethanol for 30 s. Next, the slides were dehydrated in the following solutions for 30 s each: 75% ethanol, 95% ethanol, and 2 × 100% ethanol. After dehydrating, all sample slides (fluorescent or cresyl stained) were dipped in xylene to remove residual ethanol and then incubated in an additional clean xylene solution for at least 5 min when using the PixCell instrument or no more than 3 min when using the CellCut instrument. When using the PET slides with the CellCut instrument, longer xylene incubation times compromised the foil on the slide frame. Subsequent slides were processed in the same way until the target region was completely sectioned. All solutions were freshly prepared in RNase-free slide dishes (Tissue-Tek) with 200 ml of each solution.

### LCM

#### Arcturus PixCell IIe

Slides were removed one at a time from xylene and air-dried in a fume hood for 5 min before capturing the target region using a PixCell IIe LCM instrument (Arcturus/Molecular Devices) equipped with an IR laser and an inverted epifluorescence microscope. The CapSure HS LCM caps (Life Sciences, Cat # LCM0213) were used for collecting each region separately. For the microdissection of dorsal hippocampal CA2, approximately 20 slides (five sections per slide) were generated and captured over 2 days from the left hemisphere of each mouse (*N* = 3 mice). Approximately, 10 sections (2 slides) were harvested on one CapSure HS LCM cap per region for a maximum microdissection time of 30 min. Immediately after LCM, the cap was placed on the ExtracSure device in a CapSure HS Alignment Tray. Lysis was performed using either the PicoPure or QIAGEN kits as described below. Subsequent slides were processed until the entire target region was harvested.

#### MMI CellCut

Slides were removed from xylene and air-dried in the hood for at least 5 min before capturing the target region using the CellCut LCM system (Molecular Machines and Industries, MMI) equipped with an UV laser and an inverted epifluorescence microscope. PET slides were inverted and placed onto a glass slide so that the tissue section was sandwiched between the membrane and glass slide. Each target region was collected using 0.5 ml MicroDissect caps (ASEE, Cat# ST-LMD-M-500) as described for the PixCell instrument. Immediately after LCM, lysis was performed using either the PicoPure or QIAGEN kits as described below. Subsequent slides were processed until the entire target region was captured.

#### QIAGEN Lysis and RNA Isolation

Ten microliter RLT lysis buffer with ß-ME from the RNeasy^®^ Micro kit (QIAGEN, Cat #74004) was added directly on to the cap. A new RNase-free 0.5 ml microcentrifuge tube was placed onto the CapSure ExtracSure assembly and the set-up incubated at room temperature for 5 min. The microcentrifuge tube was spun with the CapSureExtracSure assembly at 800× *g* for 2 min to collect the cell extract into the microcentrifuge tube. Cell extracts were immediately frozen on dry ice. Lysate samples were stored at −80°C until RNA isolation. For RNA isolation, samples were thawed at room temperature and samples from one region were pooled into one lysate in an RNase-free 1.5 ml microcentrifuge tube. One volume of freshly prepared RNase-free 70% ethanol was added to the lysate and total RNA purification was performed by following RNeasy^®^ Micro user guide (QIAGEN, Cat #74004). RNase-free DNase set (QIAGEN, Cat#79254) was utilized to remove genomic DNA that can interfere with downstream applications. One sample was processed at a time to limit RNA degradation.

#### PicoPure Lysis and RNA Isolation

Ten microliter XB extraction buffer from the Picopure RNA Isolation kit (Thermo Fisher Scientific, Cat #KIT0204) was added into the buffer well. An RNase-free 0.5 ml microcentrifuge tube was placed onto the CapSureExtracSure assembly and incubated for 30 min at 42°C. After incubation, the CapSureExtracSure assembly with the microcentrifuge tube was spun at 800× *g* for 2 min to collect cell extract into the microcentrifuge tube. Cell extracts were immediately frozen on dry ice. Lysate samples were stored at −80°C until RNA isolation. For RNA isolation, conditioning Buffer (CB; 250 μL) was added to the PicoPure purification spin column and incubated for 5 min at room temperature. The purification spin column was then spun in the provided collection tube at 16,000× *g* for 1 min. Microdissected samples from one region were thawed and pooled into one lysate in an RNase-free 1.5 ml microcentrifuge tube and mixed with one volume 70% ethanol (supplied). The lysates were loaded onto the spin column and total RNA purification was performed by following PicoPure^®^ RNA isolation kit user guide (Thermo Fisher Scientific, Cat #KIT0204), including on-column DNase-treatment (QIAGEN, Cat#79254). One sample region was processed at a time to limit RNA degradation.

#### Assessment of RNA Quality and Quantity

Total RNA samples were analyzed for RIN and concentration using the 2100 Agilent Bioanalyzer instrument and RNA 6000 Pico assays (Agilent, Cat #5067-1513). Statistical tests comparing RNA quality and yield were performed using Graphpad Prism (v7) software (RRID:SCR_002798). Normality (KS normality test) and variances were tested prior to two-way analysis of variance (ANOVA) analyses (alpha 0.05).

### RNA-Seq Library Generation

#### Clontech SMARTer Library Generation

RNA samples (3.0 ng total RNA per sample, max input 4 μl) were depleted of rRNA sequences using the Clontech RiboGone mammalian kit according to manufacturer’s instructions. Note that we also tested the Illumina/Epicenter Ribo-Zero rRNA Removal Kit for mouse using the Clontech product note for truly low-input RNA samples but were unsuccessful in obtaining sufficient yield after the protocol. Thus, we prepared stranded RNA-Seq libraries from the RiboGone depleted RNA samples using the Clontech SMARTer Stranded RNA-Seq Kit according to manufacturer’s instructions using 20 cycles of PCR to amplify the final library. Twenty cycles of PCR were required achieve sufficient library concentration for quantitation and sequencing.

#### NuGEN Ovation Library Generation

Stranded RNA-Seq libraries were prepared using 3.0 ng total RNA per sample (max input 5 μl) with the NuGEN Ovation RNA-Seq Systems 1–16 for Model Organisms (mouse) according to manufacturer’s instructions. In this kit, rRNA is depleted prior to library amplification instead of prior to cDNA synthesis. This yield saving step allows for only 16 cycles of PCR to be sufficient when amplifying the final library. We compared 16, 18 and 20 cycles of PCR using LCM CA2 RNA samples from a separate cohort of *N* = 3 mice. The RNA was extracted using the QIAGEN kit. We found no differences in any parameters tested (number of genes, GC content, % multi-mapped). To directly compare across kits, however, we used 20 cycles of PCR. Further, the cDNA samples used for the kit comparison were not fragmented using the Covaris S series ultrasonicator, whereas the three CA2 LCM samples were fragmented by ultrasonication. This step is recommended to reduce the amount of rRNA mapped reads and to ensure equal 5′ to 3′ read distribution. Lastly, Covaris fragmentation is recommended if sequencing on the Illumina NextSeq500 system because it is optimal for libraries less than 400 nucleotides (nt). Because smaller sized libraries are recommended for the NextSeq500 instrument, fewer total reads were acquired for the NuGEN samples (average library ~500 nt) than for the Clontech samples (average library ~200 nt) during multiplexed sequencing (see below).

### NextGen Sequencing and Analyses

Libraries were analyzed for size and concentration using the 2100 Agilent Bioanalyzer instrument and the High Sensitivity DNA assay (Agilent, Cat # 5067-4626). Libraries prepared from the Clontech SMARTer and NuGEN Ovation kits were four-plexed (2 samples each, QIAGEN and PicoPure) or three-plexed (3 CA2 LCM samples) and run on a mid-level NextSeq500 lane acquiring 100 bp paired-end reads. The data are available on Gene Expression Omnibus (GEO) #GSE95257. All code used for generating the data is available upon request.

Reads were trimmed using Sickle (v1.33; Joshi and Fass, 2011) and only paired reads with a quality score >20 and a minimum length of 20 bp were aligned to the GENCODE (M9) gene models and the mm10 mouse genome assembly index using STAR (v 2.4.1d) with the recommended ENCODE RNA-Seq parameters (ENCODE Project Consortium, 2012). Transcript abundance by gene and/or exon was quantified using FeatureCounts (v1.5.0-p1; Liao et al., 2014) given GENCODE (M9) gene models. The percentage of reads aligned to exonic, intronic or intergenic features was determined using FeatureCounts and relevant GTF files derived from GENCODE (M9). Samples were normalized with DESeq2 (Love et al., 2014) using total read counts mapped to annotated genes and/or exons. Ribosomal and mitochondrial genes were excluded from analyses as one or both of these classes of transcripts were depleted during library generation. The counts by gene data were used to determine the number of genes detected above threshold. The counts by exon data were used to determine the number of exons detected. Correlation and gene coverage plots were generated using log2 normalized counts with R (v3.3.0) and deepTools (Ramírez et al., 2014), respectively. Gene coverage profiles include the top 4000 expressed genes excluding rRNA and mitochondrial genes encoded on chromosome M. For the gene coverage heatmaps data were scaled to standard deviation. Example gene loci were produced from the UCSC Genome Browser using non-normalized BAM files. The read depth for each sample is auto-scaled per sample except in Figure 4C to visualize noise.

**Figure 2:**
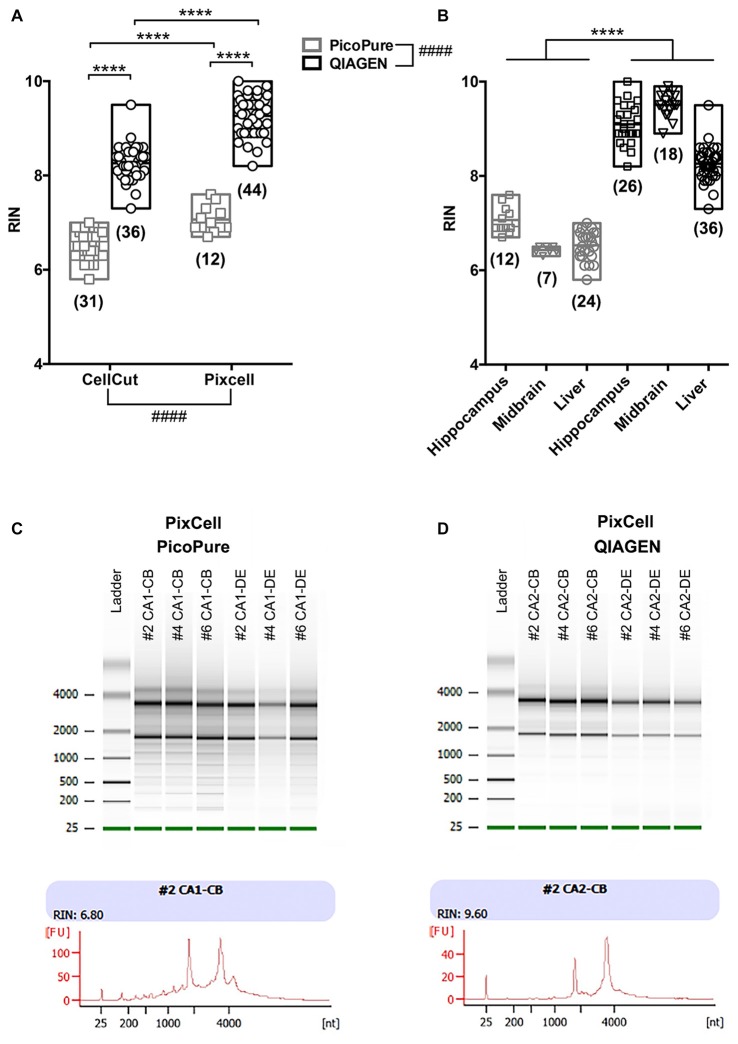
Comparison of RNA quality using different LCM methods. **(A)** Graph comparing RNA quality (RIN) from LCM RNA samples captured using the MMI CellCut or Arcturus PixCell Instrument and extracted with either the Arcturus PicoPure Isolation kit or QIAGEN Micro RNeasy kit. An overall significant effect was found for both conditions using a two-way analyses of variance (ANOVA; CellCut vs. PixCell *F*_(1,119)_ = 114.6; PicoPure vs. QIAGEN *F*_(1,119)_ = 732.5). Although, it is important to note that two groups (Pixcell PicoPure and CellCut QIAGEN) were solely represented by one tissue type (see Experimental Summary in Table 1). There was also a significant interaction between the two conditions (Interaction *F*_(1,119)_ = 9.177, *p* = 0.003). **(B)** The same data shown in A plotted by tissue type. Each tissue (Hippocampus, Midbrain and Liver) showed a significant increase in RIN with the QIAGEN kits vs. PicoPure kits using Sidak’s multiple comparisons *post hoc* test. All data were normally distributed (passed KS normality test) and had similar variances as tested by Brown-Forsythe test. **(C,D)** Representative Bioanalyzer gel (top) and electropherogram traces (bottom) from PixCell LCM RNA samples extracted using either the **(C)** Arcturus PicoPure Isolation kit or **(D)** QIAGEN Micro RNeasy kit. Note that these LCM samples were acquired simultaneously from different brain regions (CA1 vs. CA2) on the same sections from three mouse brains (#2, #4 or #6). Graphs are plotted min to max with a line at the mean. Numbers in parentheses indicate technical replicates. ^####^Overall group effect; ****post hoc result *p* < 0.0001; CB, cell body; DE, dendrite.

**Figure 3:**
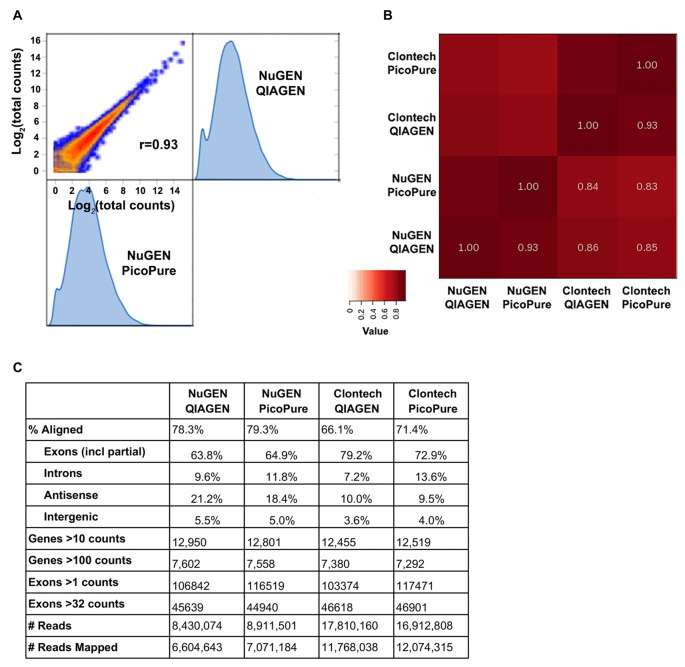
Sequencing metrics comparing library kits and RNA quality. **(A)** Log2 expression correlation plot of NuGEN libraries made from high (QIAGEN) and low (PicoPure) quality RNA. **(B)** Heat map summary of correlations from NuGEN and Clontech libraries. Note that the largest variation is from the library kit and not RNA quality. **(C)** Table of sequencing metrics. Note that due to their larger library sizes, NuGEN samples obtained fewer total reads than Clontech. Data were normalized for read depth prior to gene and exon analyses.

**Figure 4:**
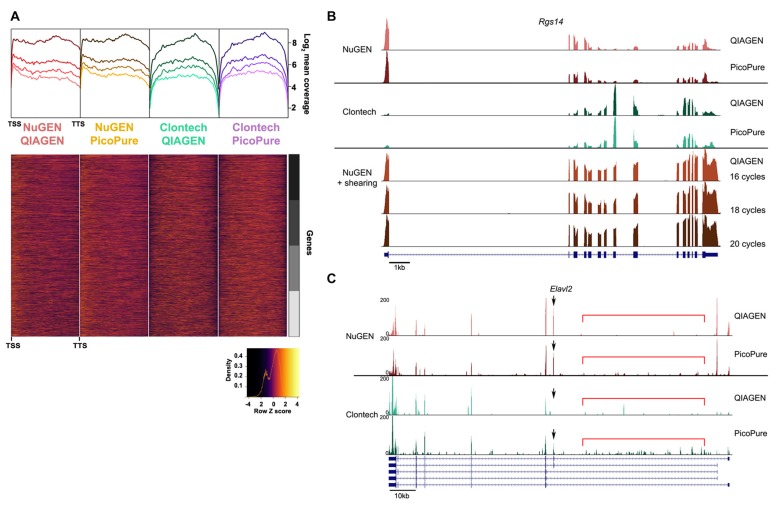
Comparison of gene coverage. **(A)** 5′-3′ average gene coverage for each sample. (Top) Gene coverage is broken down by expression with the darker colors corresponding to the highest expressing genes. (Bottom) Scaled heat map of the gene coverage graphed above. Note that libraries made with the NuGEN kit have greater gene coverage at the 5′ and 3′ ends compared to libraries made with the Clontech kit. **(B,C)** Representative gene loci to visualize read coverage from each sample. **(B)** *Rgs14* locus illustrates the greater 5′ and 3′ end coverage in the NuGEN samples. The 5′ bias in the NuGEN samples is remedied by shearing. NuGEN gene coverage is unaltered by increasing cycles of PCR. **(C)** *Elavl2* locus illustrates the increased intronic read coverage (denoted by red bar) seen in PicoPure or low quality samples. Differential isoform detection is denoted with a black arrow.

#### Cross-Correlation with Published Datasets

Comparisons with Hipposeq (Cembrowski et al., 2016) data (NCBI GEO accession no. GSE74985) were performed in R using log_2_ median normalized counts from CA2 samples (*N* = 3 for both datasets). FASTQ files were run through identical pipelines as described above with the following changes; the GTF file was derived from vM12 GENCODE gene models, the Hipposeq dataset was aligned with STAR using single-end and unstranded parameters, and no genes were excluded from analyses. Approximately 22 million reads were mapped from the Farris dataset (76% of total aligned reads were assigned to genes) and 32 million reads from the Hipposeq dataset (62% of total aligned reads were assigned to genes). The correlation plot consists of the mean per dataset from 14,045 genes with at least one count in all replicates of both datasets. The MA plots were centered by the mean of each dataset and one representative sample from each dataset is shown. The coverage profiles and heatmaps were generated as described above.

## Results

### Type of Laser and RNA Extraction Method Impact RNA Quality

In order to determine the optimal workflow for LCM RNA samples for downstream RNA-Seq we compared the RNA quality of LCM samples (as measured by RIN) captured from various mouse brain and liver samples (Table 1) using two common LCM instruments, the MMI CellCut and Arcturus PixCell IIe, which differ in the type of laser used (UV and IR, respectively) and method of capturing tissue (polymer slides vs. polymer-coated caps, respectively). We also tested two commercially available methods for RNA extraction for performance on RNA quality and quantity, the Arcturus PicoPure isolation kit (current standard in LCM) and the QIAGEN RNeasy micro kit (Figure 1A). These kits differ in the recommended time and temperature of tissue lysis with the PicoPure lysis incubation being 30 min at 42°C and the QIAGEN lysis incubation conditions being 5 min at room temperature. Furthermore, for a within sample experiment to compare the RNA extraction kits, we simultaneously captured two adjacent brain regions, hippocampal subregions CA1 and CA2 (Figures 1B,C), using the PixCell IIe instrument and isolated RNA using either the PicoPure (CA1 samples) or QIAGEN (CA2 samples) kits.

**Table 1:** Experimental summary of laser capture microdissection (LCM) samples.

Condition	# Experimental replicates^1^	# Biological replicates^2^	# Technical replicates^3^	Sample total
PixCell QIAGEN	7	15	2–8	44
PixCell PicoPure	2	6	2	12*
CellCut QIAGEN	4	18	2	36**
CellCut PicoPure	6	17	1–2	31
OIAGEN				
Hippocampus	4	6	2–8	26
Midbrain	3	9	2	18
Liver	4	18	2	36**
PicoPure				
Hippocampus	2	6	2	12*
Midbrain	2	5	1–2	7
Liver	4	12	2	24

^1^# of separate RNA extraction experiments; ^2^# of different animals; ^3^# of regions captured per biological replicate. *,**Denotes same samples.

When comparing the CellCut vs. the PixCell LCM instruments, we found that the PixCell instrument produced samples with significantly higher quality RNA (Figure 2A, *p* < 0.0001, CellCut vs. PixCell *F*_(1,119)_ = 114.6, two-way ANOVA). This was true when using either extraction kit, however, we note that our PixCell PicoPure samples consisted solely of samples captured from brain tissue and our CellCut QIAGEN samples were entirely from liver tissue (See Table 1).

When comparing the PicoPure vs. QIAGEN RNA extraction kits, RNA extracted using the QIAGEN kit produced LCM samples with remarkably higher quality RNA compared to the PicoPure kit (Figure 2A, *p* < 0.0001, PicoPure vs. QIAGEN *F*_(1,119)_ = 732.5, two-way ANOVA). This was true across numerous experimental and biological replicates (Table 1) for samples captured from multiple brain regions and the liver (Figure 2B, Hippocampus, Midbrain and Liver *p* < 0.0001, one-way ANOVA, Sidak’s *post hoc* test). The difference in RNA quality can clearly be seen in the representative bioanalyzer gels and electropherograms in Figures 2C,D, where LCM samples extracted with the QIAGEN kit (CA2) have cleaner gels with less degradation products compared to adjacent samples extracted with the PicoPure kit (CA1). In these samples, tissue from two adjacent brain regions (CA1 vs. CA2) was captured using the PixCell instrument and extracted using either the QIAGEN (CA2 samples) or PicoPure (CA1 samples) kits from three biological replicates. Approximately 75 eight-micron sections were captured per region per animal (Table 2). The RNA yield/area ratio from QIAGEN samples was not significantly different from PicoPure samples for either cellular compartment, cell body (CB) or dendrites (DE; Table 2; PicoPure vs. QIAGEN, *p* = 0.0925, *F*_(1,4)_ = 4.845, two-way ANOVA). Note that the anatomical area of the CA2 region is smaller than the anatomical area of the CA1 region (See Figure 1C) providing lower yields, but similar area/yield ratios (Table 2). These within-sample data prove that the QIAGEN kit yields superior RNA quality of comparable concentration.

**Table 2:** Within sample comparison of RNA quality from PixCell laser captured tissues.

		QIAGEN
Sample	# of sections	Area size (mm^2^)	Yield (ng)	Yield/Area	RIN
#2 CA2-CB	72	0.9	11.86	13.18	9.6
#4 CA2-CB	78	1.2	19.5	16.25	9.6
#6 CA2-CB	77	1	16.24	16.24	10
#2 CA2-DE	71	2.9	3.24	1.12	9.5
#4 CA2-DE	77	2.9	3.59	1.24	9.7
#6 CA2-DE	76	3.8	3.49	0.92	9.3
		**PicoPure**
#2 CA1-CB	74	2.8	54.89	19.60	6.8
#4 CA1-CB	70	3	49.87	16.62	7.1
#6 CA1-CB	72	2.6	55.84	21.48	6.7
#2 CA1-DE	80	5.7	9.39	1.65	7.3
#4 CA1-DE	72	5.9	4.75	0.81	7.6
#6 CA1-DE	76	7.4	8.55	1.16	7.5

### NuGEN Ovation RNA-Seq System Is Ideal for Low-Input Samples

Next we compared two commercially available RNA-Seq library generation kits to determine which is optimal for low-input samples. We tested the Clontech SMARTer stranded RNA-Seq kit and the NuGEN Ovation RNA-Seq system for mouse. Both kits use total RNA as input material (10–100 ng total RNA recommended), use random hexamer primers to generate cDNA (the NuGEN kit also uses poly-dT primers) and maintain strandedness. The kits differ in their methods to remove rRNA, with the Clontech kit removing rRNA prior to cDNA synthesis and the NuGEN kit depleting rRNA prior to library amplification. The kits also differ in their chemistry and length of protocol, with the Clontech kit taking less than 1 day and the NuGEN kit requiring 2 days to generate libraries for sequencing.

We were able to successfully generate cDNA libraries from both kits using 3 ng total RNA input (Figure 3). We compared samples generated from both high quality (QIAGEN, RIN 9–10) and low quality (PicoPure, RIN 6–7) RNA and found that the RNA-Seq data were more strongly correlated when samples were generated with the same library kit (0.93) than when using the same input RNA (0.83–0.86), indicating that the library chemistry introduces more variation in sequencing samples than RNA quality (Figures 3A,B). Additionally, the high-quality RNA samples correlated with each other slightly better than the low-quality RNA samples correlated with each other (0.86 vs. 0.83), although more samples are needed to test whether this effect, if any, is significant. We also found a higher percentage of reads mapped to the mouse genome from samples made with the NuGEN kit compared to samples made with the Clontech kit (Figure 3C, 78.3 and 79.3% vs. 66.1 and 71.4%), although NuGEN samples acquired fewer overall reads (more on this below). Furthermore, fewer reads mapped to introns and more genes were detected with greater read depth with the high-quality RNA samples (Figure 3C).

The fact that about half as many reads were acquired with the NuGEN samples can likely be attributed to the fact that we omitted the cDNA shearing step that would have resulted in NuGEN cDNA libraries within the preferred range (<400 bp) of the NEXTseq instrument (Clontech cDNA libraries are ~200 bp). The lack of shearing also resulted in a slight 5′ bias in read coverage for NuGEN samples (Figure 4A). However, we detected more coverage at both the 5′ and 3′ ends for NuGEN samples compared to the Clontech samples. This was true for both high and low expressing transcripts (Figure 4A). Subsequent studies confirmed that including the cDNA shearing step corrects the 5′ bias (example locus shown in Figure 4B). Furthermore, despite the fewer number of reads acquired, we detected a similar number of genes and exons with both kits, indicating that the NuGEN samples had sufficient coverage with less read depth that could ultimately lead to cost savings when considering how deep to sequence.

Lastly, likely due to the differences in the rRNA depletion methods, the NuGEN kit generated libraries of suitable concentration for sequencing with fewer PCR cycles than the Clontech kit (16 cycles vs. 20 cycles). This is important because fewer PCR cycles minimizes the amount of amplification bias in the resulting libraries providing a more faithful representation of the starting material. Using the RiboGone rRNA depletion kit prior to cDNA synthesis in the Clontech kit likely results in a loss of yield that requires an increased number of PCR cycles to achieve sufficient cDNA concentration for sequencing. Note that Clontech has since introduced a library generation kit (SMARTer Stranded Total RNA-Seq Kit- Pico Input) that depletes rRNA prior to library amplification similar to the NuGEN kit but with SMARTer chemistry. As of yet we have not compared performance of this new kit with those tested here.

### Optimized LCM-RNA-Seq Method Improves Variability and Gene Coverage Compared to Current Field Standards

In order to assess how our methods compared to the currently employed methods for obtaining RNA-Seq data from small samples, we cross-correlated our hippocampal CA2 LCM-RNA-Seq data (*N* = 3 mice) to a commendable recent RNA-Seq study that used manual sorting of fluorescently-labeled mouse hippocampal neurons, including area CA2 (*N* = 3 mice), from microdissected slices for RNA-Seq (Cembrowski et al., 2016). Notably, Cembrowski et al. (2016) isolated total RNA using the PicoPure RNA isolation kit, which we report produces significantly lower quality RNA than the QIAGEN RNeasy kit. However, the RIN values for their samples were not reported for us to compare. In line with common practice, they generated amplified cDNA prior to library generation using the NuGEN Ovation RNA-Seq V2 kit. Compared to the NuGEN kit used in our study, the V2 kit similarly uses total RNA as input, as well as random and poly dT priming, and does not deplete the samples of rRNA prior to cDNA synthesis. In contrast, the V2 kit amplifies the cDNA using isothermal amplification and does not retain strandedness or deplete rRNA prior to sequencing. We believe these differences might lead to fewer reads aligned per gene at a given sequence library depth. Furthermore, we acquired paired-end reads, while Hipposeq acquired single-end reads.

**Figure 5:**
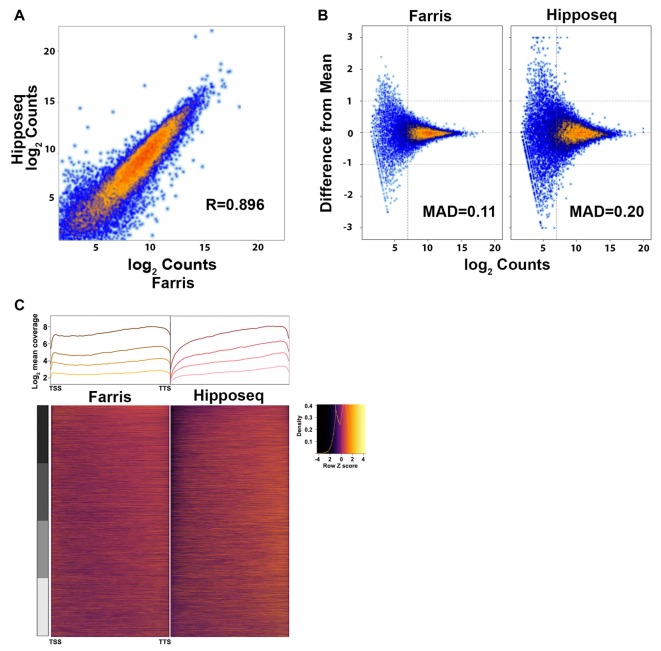
Cross-correlation of RNA-Seq expression data. **(A)** Correlation plot of the CA2 RNA-Seq mean expression data presented here (*N* = 3) vs. the CA2 RNA-Seq data published in Cembrowski et al. (2016) referred to here as Hipposeq (http://hipposeq.janelia.org, *N* = 3). **(B)** MA plots of a representative sample from each dataset depicting the mean absolute deviation (MAD) vs. abundance. The threshold for noise is drawn as a vertical line. **(C)** 5′-3′ average gene coverage for each dataset. (Top) Gene coverage is broken down by expression with the darker colors corresponding to the highest expressing genes. (Bottom) Scaled heat map of the gene coverage graphed above.

The two datasets correlated with an *R* = 0.896 using the average log2 counts from genes detected in both datasets, indicating that the majority of genes were highly correlated across datasets (Figure 5A). However, our data set showed less variability between replicates compared to the Hipposeq dataset as evinced by the lower mean absolute deviation (MAD; Figure 5B). Furthermore, our data produced more uniform coverage across gene transcripts compared to the Hipposeq dataset, which had a slight 3′ bias (Figure 5C).

In terms of genes detected, the majority of genes were shared between datasets. However, approximately 1000 genes were detected in the Hipposeq dataset that were not detected in our dataset and approximately 1700 genes detected in our dataset that were not detected in the Hipposeq dataset. The distribution of counts for these genes was generally below the level of noise (see Figure 5B) indicating transcripts with low copy numbers. Furthermore, it is likely that a subset of the genes detected in our dataset, but not the Hipposeq dataset, came from contaminating cell types and their projections in the CA2 GFP+ region we captured. Taken together, the data suggest that the methods we optimized for low-input RNA-Seq, namely the RNA extraction and library generation protocols, produce sequencing results with lower variability across replicates and more uniform gene coverage. Thus, our method should yield greater statistical power in detecting differentially expressed transcripts.

## Discussion

High fidelity transcriptomes of specific cell types are required for complete biological understanding of complex tissues. While sequencing technologies are quickly emerging to deal with both low quality and/or low quantity RNA, in circumstances where high quality RNA can be obtained, the resulting sequencing metrics are superior (Adiconis et al., 2013; Gallego Romero et al., 2014). We identified two LCM parameters that significantly affect RNA quality, the LCM laser/instrument and the RNA isolation kit. We show that for small, thinly sectioned, fresh-frozen mouse tissues, IR-based LCM instruments produce modestly higher quality RNA than UV-based LCM instruments. We also show that the QIAGEN RNA extraction kit produces significantly higher quality RNA than the PicoPure RNA isolation kit, the current standard in the field for acquiring RNA from small inputs. We further show that this high-quality RNA produces better sequencing metrics using two commercially available low-input RNA-Seq library kits. Lastly, we identified the NuGEN Ovation RNA-Seq kit as the optimal choice for low-input samples due to its minimal requirement of PCR amplification and even distribution of read coverage.

The type of LCM instrument chosen for a given experiment likely depends upon what instrument and resources are available. However, given the option between the two systems, our data suggest that the IR-based instrument outperforms the UV-based instrument in terms of producing higher RINs for small, thinly sectioned fresh frozen tissues. This is most likely due to the tissue damage and resulting RNA degradation that occurs when using the UV laser, which has a larger impact on small target areas. However, we note that two out of the four comparison groups (see Table 1) contain samples from only one tissue type, brain or liver. Thus, it is possible that some of the effect of LCM instrument may be driven by tissue type and the different levels of RNases within. For brain, when comparing a subset of the data to test the effect of LCM instrument, we still detect a statistically significant difference in RIN values (*p* < 0.01, PixCell Hippocampus PicoPure RIN mean = 7.07 (*N* = 12) vs. CellCut Hippocampus/Midbrain PicoPure RIN mean = 6.65 (*N* = 11), unpaired, one-tailed Mann Whitney *t*-test), indicating that, at least for brain with the methods tested here, the IR-instrument produced a modest but statistically significant increase in RNA quality. Additional experiments varying only the LCM instrument are needed to test whether this is also the case for liver. Furthermore, we found the effect of LCM instrument on RNA integrity was minimal (IR-UV RIN mean difference = 0.56 and 1.0 when isolated with PicoPure or QIAGEN, respectively) compared to the impact of RNA isolation kit (QIAGEN-PicoPure RIN mean difference = 1.76 and 2.20 for UV and IR, respectively); the interaction effect was essentially additive (IR QIAGEN-UV PicoPure RIN mean difference = 2.76). The major difference between the QIAGEN and PicoPure RNA isolation kits is the tissue lysis step. QIAGEN incubates the LCM tissue for 5 min at room temp whereas PicoPure incubates the tissue for 30 min at 42°C. In our system, the shorter incubation time in the QIAGEN kit did not significantly affect the RNA yield/area, suggesting that longer lysis incubation times negatively affect RNA quality without much gain in RNA yield. Interestingly, when comparing previous LCM articles that used either PicoPure (Butler et al., 2016; Mo et al., 2016) or QIAGEN (Wang et al., 2009; Cummings et al., 2011) kits for RNA isolation, those that used the QIAGEN kit reported higher RIN values compared to those that used the PicoPure kit, confirming that our findings are robust and translatable to many different types of samples. One study (Mo et al., 2016), also compared RNA isolated using the PicoPure kit with another RNA extraction kit, RNAqueous-Micro Kit, that reportedly had faster extraction times at a lower temperature and a smaller elution volume. However, they reported a poor RNA yield with this kit and little effect on RNA quality. The differences in our results, may be explained, in part, by differences in sample type (they used 9 μm fresh-frozen human colonic biopsies) and/or LCM instrument (they used the ArcturusXT LCM, a combined IR/UV-based instrument). Based on their use of PEN membrane slides, it can be inferred that they used the UV laser, which based on our data may have adversely affected their RNA quality. It is also possible that the QIAGEN RNeasy Micro kit would yield higher quality RNA than the RNAqueous-Micro Kit for another reason that is not immediately clear in the protocols. Nonetheless, our data clearly show that high-quality RNA, of sufficient concentration, can be obtained from minute laser captured tissue samples for subsequent sequencing applications.

Although LCM affords additional spatial information, there are certainly alternative genetic approaches for acquiring cell-type specific RNA for transcriptome analyses, with cell-type specific thiouracil (TU)-tagging (Gay et al., 2013, 2014) being one example. In this system, cell specificity is achieved by crossing a transgenic mouse that expresses cre recombinase in the cell type of interest with a transgenic mouse that expresses uracil phosphoribosyl transferase (UPRT), an enzyme that converts 4-TU to thiouridine, only in the presence of cre recombinase. This results in a system where only the cell type of interest expresses UPRT, and upon systemic administration of 4-TU can incorporate the thiouridine into newly transcribed transcripts, which can be further biochemically isolated from mixed tissues. Our optimized workflow for low-input RNA-Seq would be particularly suitable for TU-labeling experiments that seek transcriptome data from target cells with small populations and thus limited amounts of RNA.

Using library generation approaches for low-input total RNA, we were able to successfully generate cDNA libraries from both low- and high-quality RNA LCM samples. Both library generation approaches detected over 12,500 genes above the noise threshold, which is consistent with the number of genes detected by 454 sequencing of polyA selected mRNA in rat hippocampal CA1 pyramidal cells (Cajigas et al., 2012). Both the Clontech SMARTer and NuGEN Ovation kits afford a number of advantages for working with minimal amounts of total RNA. First, the kits use random primers (the NuGEN kit also uses poly dT primers) to ensure partially-degraded RNA, noncoding RNA and mRNA are included in the library. Second, both kits preserve strand information, which enables more accurate determination of isoform specific expression levels and supports discovery of novel transcripts. Furthermore, in the NuGEN kit, the samples are not depleted of rRNA prior to first strand synthesis, which minimizes initial loss of RNA yield. This increase in library yield minimizes the number of PCR cycles required for low-input samples (16 PCR cycles vs. 20 PCR cycles) thereby decreasing the PCR bias and duplication levels in the libraries. It is also possible that retaining rRNA during library generation may functioning as “carrier RNA” to enhance RNA recovery by minimizing target nucleic acid absorption onto microtube walls. We note that Clontech has since introduced a library generation kit (SMARTer Stranded Total RNA-Seq Kit- Pico Input) that depletes rRNA prior to library amplification similar to the NuGEN kit but with SMARTer chemistry. We have not tested this kit but based on our results, we would predict this kit to require fewer PCR cycles than the kit we tested using the same amount of input.

When comparing the sequencing data from the libraries generated from both kits using low and high-quality RNA, we found that the library generation method had the largest effect on reproducibility. Libraries made from the same input RNA correlated with each other less than libraries made from the same kit, implying the library chemistry had a larger effect on reproducibility than RNA quality. However, the high-quality RNA samples correlated with each other more than the low-quality samples, indicating that RNA quality had an effect on reproducibility using the methods here, more samples would be needed to test whether this effect, if any, is significant. We also found that the NuGEN libraries had a higher percentage of reads mapped to annotated genes and the most genes detected above noise despite having acquired fewer reads during the sequencing run for technical reasons. These data suggest that the NuGEN samples have greater coverage depth over annotated genes. Indeed, we found that the NuGEN samples produced more uniform 5′ to 3′ gene coverage, whereas the Clontech SMARTer kit samples had less coverage near both ends. Uniform gene coverage is important for detecting and quantifying differential isoform expression. In previous reports (Adiconis et al., 2013), these two kits performed similarly in terms of gene coverage. However, an important difference between the two studies is the length of sequencing reads. Here we acquired 100 bp paired-end reads, compared to 76 bp paired-end reads in Adiconis et al. (2013). We believe that the differences in library size between the two kits (200 bp for Clontech; 400 bp for NuGEN) combined with the longer sequencing length and 20 bp minimum mapping length used in our study could have negatively affected libraries with shorter read fragments and contributed to a loss of end boundaries. Researchers should keep these parameters in mind when choosing the library generation and sequencing methods most suitable for the project goals. Lastly, for both kits, the libraries made with high-quality RNA had less noise in terms of percentage of reads mapped to intronic regions. Thus, our optimized method generates high-quality RNA from minute laser captured tissue samples that significantly improves sequencing results.

The cross-correlation analysis between our CA2 RNA-Seq and the Hipposeq CA2 neuron RNA-Seq data demonstrated a high degree of correlation (*r* = 0.896), suggesting that both methods reliably detect the CA2 transcriptome. However, our replicates had less variability, which when compared to other samples should produce a greater number of differentially expressed genes. The lower variability seen in our dataset could be attributable to differences in the RNA extraction methods (QIAGEN vs. PicoPure), library generation methods (stranded vs. unstranded, rRNA depletion vs. no depletion) and/or sequence format (paired-end vs. single-end). Furthermore, our dataset had more uniform 5′-3′ coverage than the Hipposeq dataset. When adding that our libraries were strand-specific and sequenced with paired-end reads, it suggests our methods would be particularly suitable for quantification of isoform-specific expression.

Our improvements yield high-quality RNA from minute tissues that can generate highly reproducible transcriptome data. Thus, we believe our optimized methods for producing RNA-Seq data from minute tissue samples will greatly enhance the robustness of RNA-Seq datasets in the field.

## Author Contributions

SF, YW, JMW and SMD designed research. SF and YW performed research. SF and JMW analyzed data. SF and SMD wrote the article.

## Conflict of Interest Statement

The authors declare that the research was conducted in the absence of any commercial or financial relationships that could be construed as a potential conflict of interest.
